# Association between motor and math skills in preschool children with typical development: Systematic review

**DOI:** 10.3389/fpsyg.2023.1105391

**Published:** 2023-02-02

**Authors:** Pedro Flores, Eduarda Coelho, Maria Isabel Mourão-Carvalhal, Pedro Forte

**Affiliations:** ^1^Sports Department, Higher Institute of Education and Sciences of the Douro, Penafiel, Portugal; ^2^Sports Department, University of Trás-os-Montes and Alto Douro, Vila Real, Portugal; ^3^Research Center in Sports, Health and Human Development, Covilhã, Portugal

**Keywords:** children, preschool, motor skills, math skills, mathematical performance

## Abstract

Mathematics has been the subject in which many school-age children have revealed many difficulties. Research carried out in an attempt to understand the causes of failure in this area pointed to a positive association between mathematical performance and motor performance. Given the importance of math development in future school outcomes, knowing which specific motor components are most associated with math performance can help educators define better strategies for teaching mathematics. In this sense, the present systematic review study aimed to identify the components of motor skills most positively associated with mathematical performance in children with typical development who attend preschool. The PRISMA methodology (Preferred Reporting Items for Systematic Reviews and Meta-Analyses) was used in this study. The databases searched were ERIC, PubMED, SciELO, Scopus and Web of Science. A total of 2,909 articles were identified, of which 18 were included in this systematic review. The main results showed positive associations between fine motor skills, namely fine motor coordination and visuomotor integration, and mathematical performance. The math skill of numerical counting was the most associated with FMS. The main characteristics of the instruments used showed that the tasks of copying figures or drawings are the most used to assess visuomotor integration and the tasks of handling objects with pinch-like movements are the most used to assess fine motor coordination. Given the importance of mathematical performance in future school results, identifying early children with difficulties in fine motor skills will help educators to design better strategies for teaching mathematical skills. In this sense, the need to identify instruments to assess fine motor skills in preschool children with characteristics that facilitate their administration by the educator in the classroom context, i.e., requiring little administration time, not requiring much experience or training, the possibility of being administered to the group/class, few material resources, and the results can be easily interpreted, classified, and associated with mathematical performance.

## 1. Introduction

The relationship between motor and cognitive development goes back to Piaget’s theory of cognitive development, highlighting the interconnection between individuals and the environment. The process of assimilation and, in particular, accommodation leads to the formation of new and more complex cognitive structures when the child interacts with the environment (Piaget and Inhelder, 1966). According to Wallon (1941), children develop through movement, from action to representation, from physical to cognitive. Pelicier et al. (1996) stated that motor and psychological functions are the two fundamental elements of human behavior. Initially, they develop together, then they specialize and differentiate, although they remain subject to reciprocal interactions (Adolph and Franchak, 2017; Kim et al., 2018). The idea of “*learning to learn*” suggests that early learning is motor- system centered with brain systems involved in posture, grip, vision, and motor control, and as the child adapts to changes in cognitive and motor skills, he or she develops simultaneously (Adolph, 2005). Today, there is neurophysiological and neuroimaging evidence that the prefrontal cortex, cerebellum and connection structures are co-active in certain cognitive and motor tasks suggesting an interrelationship between motor and cognitive development (Diamond, 2000; Abe and Hanakawa, 2009).

Cross-sectional research has shown positive associations between motor skills and cognitive and academic assessments (Kantomaa et al., 2013; Lopes et al., 2013; Haapala et al., 2014; Diamond, 2015; Geertsen et al., 2016). Additionally, longitudinal studies carried out in typically developing populations have found a relationship between motor development and cognitive development throughout the life cycle. Motor skills acquired at a very early age may be related to cognitive skills during childhood (Michel et al., 2016), adolescence (Cantell et al., 2003), and even adulthood (Kuh et al., 2006). Recent studies have highlighted that motor skills influence academic performance in the early years (Carson et al., 2016; Alvarez-Bueno et al., 2017; Zeng et al., 2017; Macdonald et al., 2018; De Waal, 2019; Duncan et al., 2019; Haapala et al., 2019; Malambo et al., 2022), and are described as one of the criteria for school readiness (Department for Education, 2020; Jones et al., 2021). For this reason, an assessment of motor skills at an early age can help to identify children who are likely to perform poorly in academic skills in advance (Roebers et al., 2014; Cameron et al., 2016; Pitchford et al., 2016; Schmidt et al., 2017; Goodway et al., 2019).

### 1.1. Motor skills

The term fundamental motor skill was defined by Wickstrom (1977) as a basic motor activity for more advanced and highly specific activities such as running, jumping, and throwing, among others. Currently, the term fundamental motor skill reflects various terminologies that have been used in the literature such as motor proficiency, motor performance, fundamental movement ability, fundamental motor skill, motor skill and motor competence (Robinson et al., 2015).

Various terms have been used to describe fundamental motor skills, such as: gross motor skills (Pang and Fong, 2009; Mostafavi et al., 2013), fundamental motor patterns (Barnett et al., 2012), and fundamental movement skills (Staples and Reid, 2010; Barnett et al., 2015). Similarly, various terminologies have been commonly used to describe fine motor skills, such as: fine motor proficiency, fine motor accuracy, fine motor integration, manual dexterity, or fine motor coordination (Bruininks and Bruininks, 2005) or performance motor skills (Sortwell et al., 2022).

In view of the numerous terminologies found in the literature, the present systematic review is based on the terminologies used in the reviews published by Macdonald et al. (2018), Donnelly et al. (2016) and van der Fels et al. (2015), due to the fact that they present similar objectives. These reviews used the term Motor Skills and the underlying domains as gross motor skills and fine motor skills.

Motor skills (MS) refer to efficient and effective actions resulting from a learning process (Magill, 1984). According to movement control and precision, they are divided into two categories, gross motor skills (GMS) and fine motor skills (FMS). These two categories of motor skills are used in research to analyze the relationship between cognitive and motor development (Grissmer et al., 2010; Gentier et al., 2013; Raisbeck and Diekfuss, 2015; Van der Fels et al., 2015; Oberer et al., 2017; Macdonald et al., 2018; Haywood and Getchell, 2019).

GMS primarily use movements produced by large muscle groups. They include motor skills that imply movement of the body in space, such as walking, running, jumping, sliding, and postural skills, which refer to the ability to keep a controlled position or posture during a specific task or activity (they can be dynamic or static) and manipulative skills used to control objects in actions such as grabbing, hitting, absorbing, lifting, etc., with hands, feet or using other objects for that purpose (Ulrich, 2000; Grissmer et al., 2010; Lopes et al., 2013; D’Hondt et al., 2014; Magistro et al., 2015; Rudd et al., 2015; Chang and Gu, 2018; Haywood and Getchell, 2019). On the other hand, FMS can be defined as movements produced by small muscle groups that involve activities with great precision, implying two distinct capabilities, motor coordination, and visual integration, as well as the integration of both (Carlson et al., 2013). In this context, different types of FMS can be identified depending on the capabilities involved (Kimmel and Ratliff-Schaub, 2011; Carlson et al., 2013). A type of FMS consists of fine motor coordination (FMC), which refers to movements that involve oculus-manual coordination (eye-hand), manual dexterity, motor sequencing, and speed and precision, such as tracing, touching with fingers, building with Legos/blocks, moving coins from one place to another or inserting them into a slot, etc., which can also be called non-graphomotor skills (Davis and Matthews, 2010; Suggate et al., 2018).

**Figure 1:**
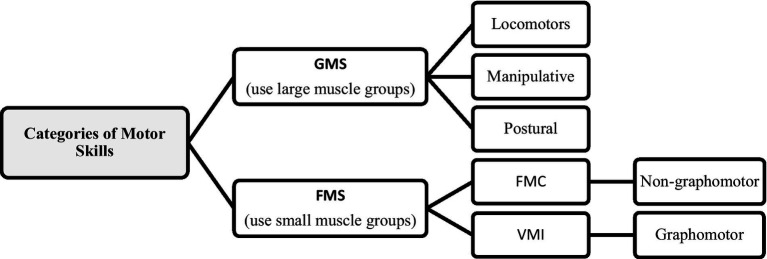
Summary of motor skills categories (Author’s figure).

Another type of FMS consists of visual motor integration or visuomotor integration (VMI) and spatial or visuospatial integration (VSI), which refers to the organization of small muscle movements of the hand and fingers through the processing of visual and spatial stimuli, more based on synchronized hand-eye movements (Sortor and Kulp, 2003; Carlson et al., 2013; Fuhs et al., 2014; Kim et al., 2018), typically writing, drawing, copying shapes, letters, or other stimuli (Beery and Buktenica, 1997; Grissmer et al., 2010; Roebers et al., 2014; Oberer et al., 2017) that can be called graphomotor skills (Davis and Matthews, 2010; Suggate et al., 2018). Figure 1 summarizes the categories of motor skills.

### 1.2. Mathematical performance

The performance of academic skills preferentially values two curricular areas, literacy and mathematics performance (Fernandes et al., 2016; Ribner et al., 2017). These two areas are considered prerequisites for performance in other subjects and, consequently, for academic success (Organization for Economic Cooperation and Development (OECD), 2016). However, mathematics plays an important role in the school curriculum and its development. In a modern, technological society, it is seen as a fundamental cognitive attribute, where successful early learning provides not only a framework for later learning (Duncan et al., 2007) but is also an indicator of future academic and professional success (Parsons and Bynner, 2005).

Mathematics is learned by children before school through numbers and quantities (Blair, 2002; McWayne et al., 2004) and the informal knowledge they acquire is often referred to as “basic numerical skills,” being a precondition for mathematical reasoning (Gersten et al., 2005; Jordan et al., 2006). Studies have shown that math skills in preschool education predict performance in reading, math, and science through grade 8 (children between 13 and 14 years old; Claesens and Engel, 2013).

The math skills children acquire in preschool education are important for developing a conceptual understanding of mathematics (Jordan et al., 2009; Siegler et al., 2012), as well as confidence in the ability to engage in activities that support analytical thinking, problem solving and reasoning and argumentation skills (Clements et al., 2004). However, teaching mathematics in preschool education must be related to children’s day-to-day interests, such as playing or exploring everyday situations (Silva et al., 2016).

It is recognized that pre-school children enjoy activities that develop their math skills (Ginsburg et al., 2006). However, most early childhood educators typically place greater emphasis on children’s social–emotional and literacy development and less attention to mathematics (National Research Council, 2009). Many early childhood educators avoid teaching mathematics because of their own negative experiences with it (Ginsburg et al., 2006; Clements and Sarama, 2007).

**Figure 2:**
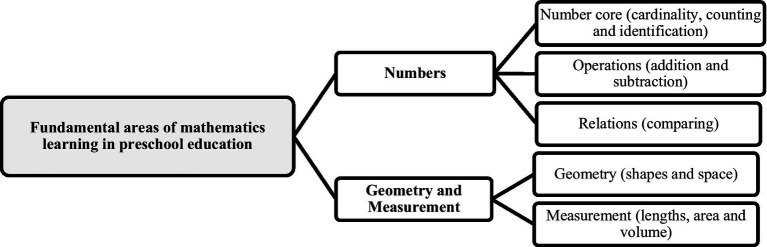
Summary of fundamental areas of mathematics learning in preschool education according to the National Research Council, 2009 (Author’s figure).

Fundamental learning for the development of mathematical competences in preschool education consists of two main areas: Numbers (1) and Geometry and Measurement (2). Each of these areas consists of different skills. The area of numbers is subdivided into the core of numbers (counting, cardinality, and identification of numbers), operations (addition and subtraction) and relations (comparing). Regarding the area of geometry and measurement, geometry consists of shapes and space, and measurements of length, area, and volume (National Research Council, 2009; Figure 2).

Numbers are abstractions that apply to a wide range of real and imaginary situations. These do not exist in isolation, but constitute a system of relations and operations by which they can be compared, added, subtracted, multiplied, and divided. These relationships apply to a wide variety of problems (National Research Council, 2009). On the other hand, geometry and measurement provide systems for describing, representing, and understanding the world. Geometry is the study of shapes and spaces (two-dimensional-2-D and three-dimensional – 3-D). Measurement is about determining the size of object shapes (National Research Council, 2009). In this sense, preschool children need to learn the following math skills (National Research Council, 2009):

**Core of numbers** (*cardinality, counting, and identification*);

*Cardinality –* Children learn the concept of cardinality when they understand that adding an object means counting to the next number (Sarnecka and Carey, 2008).

*Counting –* Counting means listing the count numbers in order, usually starting at 1. It is a way of making a 1 to 1 correspondence between each object (Wynn, 1992).

*Number Identification –* It is the ability to associate a written number (e.g., 5) with a verbal word (e.g., five; Jordan et al., 2009).

**Relationships** (*comparing*);

Higher Institute of Education and Sciences of the Douro, Sports Department, Penafiel, Portugal, Penafiel, Portugal.

*Comparing* – represents the comparison of quantities of groups of objects using words such as “more/bigger,” “less/smaller” and “equal.” A basic way to compare two quantities of objects is by direct correspondence. If a child has a noticeably larger set of black beads compared to a set of white beads, the child identifies which group has the larger amount of beads. Thus, these skills can be developed separately from other basic math skills, such as counting and cardinality and number identification, because it is not necessary to know the exact number of objects in each group to successfully compare two groups (Traverso et al., 2021).

**Operations** (*addition and subtraction*);

*Addition and subtraction –* refer to basic arithmetic skills such as adding and subtracting and are used to relate quantities. Children are only prepared to develop these skills when they understand the concepts of cardinality and counting. These skills prepare children to develop more complex arithmetic skills such as multiplication and division (Barth et al., 2008; Canobi and Bethune, 2008).

**Geometry** (*shapes and space*).

*Shapes –* Shape is the basic way children learn to name objects (Jones and Smith, 2002). Children have an innate and implicit ability to recognize and match shapes (Anderson, 2000).

*Space –* Space includes two main skills: spatial orientation and spatial visualization of images. Spatial orientation involves knowing where you are and how to move around in the world (Gelman and Williams, 1997). Children learn words such as “beside” and “between.” Later, they learn words referring to frames of reference, such as “in front of,” “behind.” The words “left” and “right” are learned much later, and are a source of confusion for several years (Gopnik and Meltzoff, 1986). In these early years, children can also learn to analyze a route through a space (Wang and Spelke, 2002). Spatial visualization of images is about understanding and performing imagined movements of 2-D and 3-D objects. This requires being able to create a mental image and manipulate it through a close relationship between these two cognitive abilities. Spatial visualization of images has been positively associated with the construction and composition of shapes (Sarama et al., 1996).

**Measurement** (*length, area, and volume*).

*Length Measurement –* quantifies the distance between points in object or space.

*Area Measurement –* is a quantity of 2-D surface area that is contained within a boundary.

*Volume Measurement –* volume introduces even more complexity by the addition of a third dimension (3-D), presenting a significant challenge to students’ spatial structuring (Curry and Outhred, 2005).

One way to more formally assess children’s understanding of measurements is through comparison tasks (Mullet and Paques, 1991).

### 1.3. Relationship between motor and math skills

The literature has pointed out a positive association between mathematics and motor skills (Macdonald et al., 2018) and, according to the theory of “*Embodied Cognition*” (Embedded Cognition), cognition emerges from the “*coupling*” (embodied relationship) of the individual with the physical and social context as a result of sensorimotor activity (Smith, 2005). According to this paradigm, body movement causes changes in neural networks, stimulating significant gains in cognition. Within this paradigm, several investigations have highlighted the importance of movement in cognition, particularly in the performance of math skills such as abstract cognitive representations in general, and in the improvement of basic numerical representations in particular (Link et al., 2013). As proposed by Fischer et al. (2018), numbers are embodied concepts and not abstractions dissociated from sensory experiences. In addition, the theory assumes that certain cognitive and motor areas of the brain are activated simultaneously when solving mathematical problems (Fischer and Brugger, 2011).

Much research has been produced in an attempt to demonstrate the association between MH and cognition, but few have considered different categories of motor skills (gross/fine) and different categories of cognitive skills (executive functions/academic success in reading and mathematics; Oberer et al., 2017). Depending on the different categories of variables studied, the results differ and it is not possible to reach conclusive data (Magistro et al., 2015; Veldman et al., 2019). Furthermore, each study analyzed different motor skills and academics, performed them in different ways, or evaluated populations with different characteristics. All these aspects contribute to a disparity of results in this area (Magistro et al., 2015; Veldman et al., 2019). It is, therefore, a complex area of study that does not allow consensual conclusions (Escolano-Pérez et al., 2020). In this sense, it is considered necessary to study the associations between the different categories of specific motor skills and academic skills, in order to contribute to the success of children’s learning.

### 1.4. Relevance of the study

The subject of mathematics was selected for this study for the following reasons: first, because it is a “universal language” (across all countries); second, because it is the subject in which many school-age children have difficulties in learning, a problem with incidence ranging from 3% to 7% (Shalev et al., 2005; Swanson et al., 2009; Devine et al., 2013, 2018). These difficulties occur more than expected (Koponen et al., 2018; Willcutt et al., 2019) and are already observed at preschool age (Desoete et al., 2012; Devine et al., 2018); third, in light of the priority for children to develop basic numeracy skills upon entry to grade 1 (Rimm-Kaufman et al., 2000; Verdine et al., 2014; Duran et al., 2018; Cameron et al., 2019; Nesbitt et al., 2019; Escolano-Pérez et al., 2020); fourth, it is a strong predictor of future academic success (Parsons and Bynner, 2005; Carr and Alexeev, 2011; Fuchs et al., 2016).

Considering the importance of mathematics in future school results, knowing which motor skills can contribute to improving mathematical performance will help educators to select and program more appropriate strategies for teaching and learning math skills.

In this sense, the present systematic review study aims to identify in children with typical development who attend preschool education, the different categories of motor skills that are associated with math skills and the instruments used in investigations carried out with this objective.

It was hypothesized that motor skills positively influence mathematical performance.

## 2. Materials and methods

### 2.1. Identification of studies

A search was carried out in electronic databases, by two reviewers (PF, PF), according to the PRISMA protocol (*Preferred Reporting Items for Systematic Reviews and Meta-Analyses*) to identify relevant studies (Moher et al., 2009). The databases searched were ERIC, PubMED, SciELO, Scopus, and Web of Science. The research was conducted on January 15, 2022 and the period was restricted to the last 10 years. The research made no restrictions regarding language. Although the last 5 or 10 years may be well-defined periods for research (Sampaio and Mancini, 2007; Donato and Donato, 2019), they may not, however, be the most appropriate. Thus, we conducted a search in the databases used in this study on systematic reviews published on the subject under study and found that the vast majority were published from 2012 onwards. Thus, the last 10 years were chosen instead of the last 5 years. EndNote was the program used to manage bibliographic references. Keywords were used to identify the relevant literature according to the objective of the study. The research was performed according to the abstract of the articles and the phrase used to detect them in all databases was: ((motor AND (motor AND (proficiency OR competency OR skill* OR development OR ability OR performance OR gross OR fine)) AND (“academic performance” OR “academic achievement” OR “academic grids” OR math* OR numeracy) AND (child* OR preschool*)).

### 2.2. Selection of studies

After removing duplicates, the titles and abstracts of the remaining studies were read, by two reviewers independently (PF, PF), and selected with reference to the predefined inclusion and exclusion criteria to assess their potential eligibility for this systematic review. Studies in which the abstract clearly indicated that they would be ineligible for inclusion were immediately eliminated, however, those in which there was some doubt as to their eligibility were kept.

Subsequently, the full texts of these articles were obtained to assess eligibility for inclusion in this review by the two reviewers (PF, PF) and in case of doubt the studies were reevaluated together, following inclusion and exclusion criteria. This research followed the PICOS criteria (population, intervention, comparison, outcome, study; Moher et al., 2009): P, preschool children with typical development; intervention, motor skills diagnosis; C, relationship between motor skills and mathematics; O, evidence of the influence of motor skills on mathematical performance; S, cross-sectional or longitudinal studies, with or without intervention, in any language and in any publication format (articles and or papers).

- Inclusion criteria:
  1. The study population should include typically developing children attending preschool education, between the ages of 2.5 and 7 years. Atypical development is defined as the development of children who exhibit early delays, deviations, or disabilities below the desired development for the same age group (Jones et al., 2014; D’Souza and Karmiloff-Smith, 2017; Johnson et al., 2021).
  2. Studies should include an association between mathematical performance and at least one specific component of motor skills (GMS and FMS; Grissmer et al., 2010; Gentier et al., 2013; Raisbeck and Diekfuss, 2015; Van der Fels et al., 2015; Oberer et al., 2017; Haywood and Getchell, 2019);
  3. The studies should be primary observational studies (longitudinal and transversal);
  4. Objective measures should be used to assess the specific components of motor skills and mathematics;
  5. Appropriate statistical analyses to report associations should include correlations.
- Exclusion criteria:
  1. Studies with populations of children diagnosed with mental illness, neurological disorders (learning difficulties, motor coordination disorder, attention deficit hyperactivity disorder, autism spectrum, and cerebral palsy), and premature children. Studies with premature children who did not develop cerebral palsy were excluded, because among the most frequent problems are those associated with GMS and FMS (de Kieviet et al., 2009; Williams et al., 2010), in which they have been shown to be a negative influence on their academic performance (McHale and Cermak, 1992; Feder et al., 2005; Feder and Majnemer, 2007; Edwards et al., 2011).
  2. Secondary studies (non-systematic and systematic reviews, with or without meta-analysis);
  3. Studies that did not show associations between math skills and at least one specific component of motor skills (Bruininks and Bruininks, 2005; Carlson et al., 2013);
  4. Studies that did not present the results aim to assess the specific components of motor skills and mathematics;
  5. Studies that did not include in statistical analyses the correlations between at least one specific component of motor skills and mathematics.

After reading the articles in full, some were excluded because they did not meet all the inclusion criteria. Eligible studies were kept and included for methodological and subsequent quality assessment, data extraction, discussion, and conclusions.

An additional search was conducted in the references of the articles included in the review in order to add relevant articles. After the search, no referenced articles met all the criteria for inclusion in this review.

### 2.3. Critical assessment of methodological quality

The methodological quality of the studies was assessed by two independent reviewers (EC and IM). In case of disagreement, the studies were jointly reassessed until a consensus was reached regarding the final score.

The Methodological Quality Checklist for studies based on Observational Methodology (MQCOM), intended for studies using observational methodology, was used to assess the methodological quality of the studies. This instrument was firstly designed by Chacón-Moscoso et al. (2018) that determined the primary criteria/dimensions to take into account when reporting research using observational methods and developed a list of metrics to quantify them (Anguera and Hernández-Mendo, 2015; Portell et al., 2015; Chacón-Moscoso et al., 2016). Recently this instrument was reduced to 16 questions (Chacón-Moscoso et al., 2019), allowing to identify the main methodological quality items needed to conduct studies based on observational methodology and offer the results as a useful tool for authors conducting studies and reviewers making publication decisions.

The MQCOM is composed of 16 items divided into 11 criteria/dimensions: Criterion 1 – Delimitation of objectives is composed of 3 items (Item 1. Reference to observational methodology, specifying whether observation is direct or indirect; Item 2. Delimitation of study objectives; Item 3. Theoretical framework referenced). Criterion/dimension 2 – Observational design, consists of 3 items [Item 4. Observation unit criteria (idiographic: study units are formed by one or more participants if there is a stable link between them; nomothetic: two or more study units); Item 5. Temporal criteria (punctual: one or two observation sessions; follow-up: more than two observation sessions); Item 6. Dimensionality criteria (one-dimensional: one level of response; multidimensional: two or more levels of response)]; Criterion/dimension 3 – Participants/observation units, consists of 1 item [Item 7. Clear specification of inclusion and exclusion criteria for observation units (reasons why some units were chosen in the study and others were not)]. Criterion/dimension 4 – Observation instruments, consists of 2 items [Item 8. Adequacy of the observation instrument (combination of field format with category system, field format, category system, or scale of estimation); Item 9. Codification manual with definition of the categories/behaviors and specification of dimensions (in multidimensional designs)]; Criterion/dimension 5 – Software use, consists of 1 item [Item 10. Software used to register data (SDIS-GSEQ v. 4.2.1./GSEQ 5, LINCE, MATCH VISION STUDIO, Transana, other: specify), control data quality (SDIS-GSEQ v. 4.2.1./GSEQ 5, LINCE, HOISAN, GT, SAS, other: specify)], and analyze data (SDIS-GSEQ, HOISAN, THEME v. 6, R, SAS, other: specify); Criterion/dimension 6 – Data, consists of 1 item [Item 11. Specification of data type as sequential/concurrent (sequential data: behaviors that cannot overlap and belong to a single dimension; concurrent data: behaviors that can co-occur and belong to several dimensions) and event-based/time-based (event-based: the primary parameter used in the record is order of events; time-based: the primary parameter is duration)]; Criterion/dimension 7 – Specification of parameters, consists of 1 item (Item 12. Type of parameters according to given use); Criterion/dimension 8 – Observational sampling, consists of 1 item (Item 13. Delimitation of sessions: clear establishment of criteria (temporal, behavioral, or mixed) for the beginning and the end of sessions within the observation period and of criteria for acceptance of sessions: between-sessions constancy, within-sessions constancy, or temporary disruptions); O Criterion/dimension 9 – Data quality control, consists of 1 item [Item 14. Between-observer reliability (agreement between the records of different observers)/within-observer reliability (agreement between the records of the same observer at two time points)]; Criterion/dimension 10 – Data analysis, consists of 1 item (Item 15. Type of data analysis performed); Criterion/dimension 11 – Interpretation of results, consists of 1 item (Item 16. In the discussion section).

According to Chacón-Moscoso et al. (2019) items are rated between zero and one. The value zero represents, does not comply; The value one represents, meets the criteria; The value 0.5 represents, partially complies. Items 3 and 15 are exceptions, as they still have different intermediate quotations between zero and one (0, 0.33, 0.67, and 1). Item 11 has a rating of nine, not applicable. The minimum value of the MQCOM is zero points and the maximum is 16 points (Chacón-Moscoso et al., 2019).

### 2.4. Data extraction

Data were independently extracted by two reviewers (PF, PF) and cross-checked, and controversial issues were discussed based on the original text to determine the final outcome. The extracted information included study characteristics: author, year, type of study and country where the study was carried out; main characteristics of the sample (number, sex and age); aim of the study, main results and conclusions; specific components of motor skills associated with different mathematical competences; instruments used to assess motor skills and mathematical competences.

Considering the methodological heterogeneity among the studies and instruments for the collection of data related to motor skills and mathematics and quality of the studies, it was not possible to carry out a meta-analysis.

## 3. Results

### 3.1. Included studies

**Figure 3:**
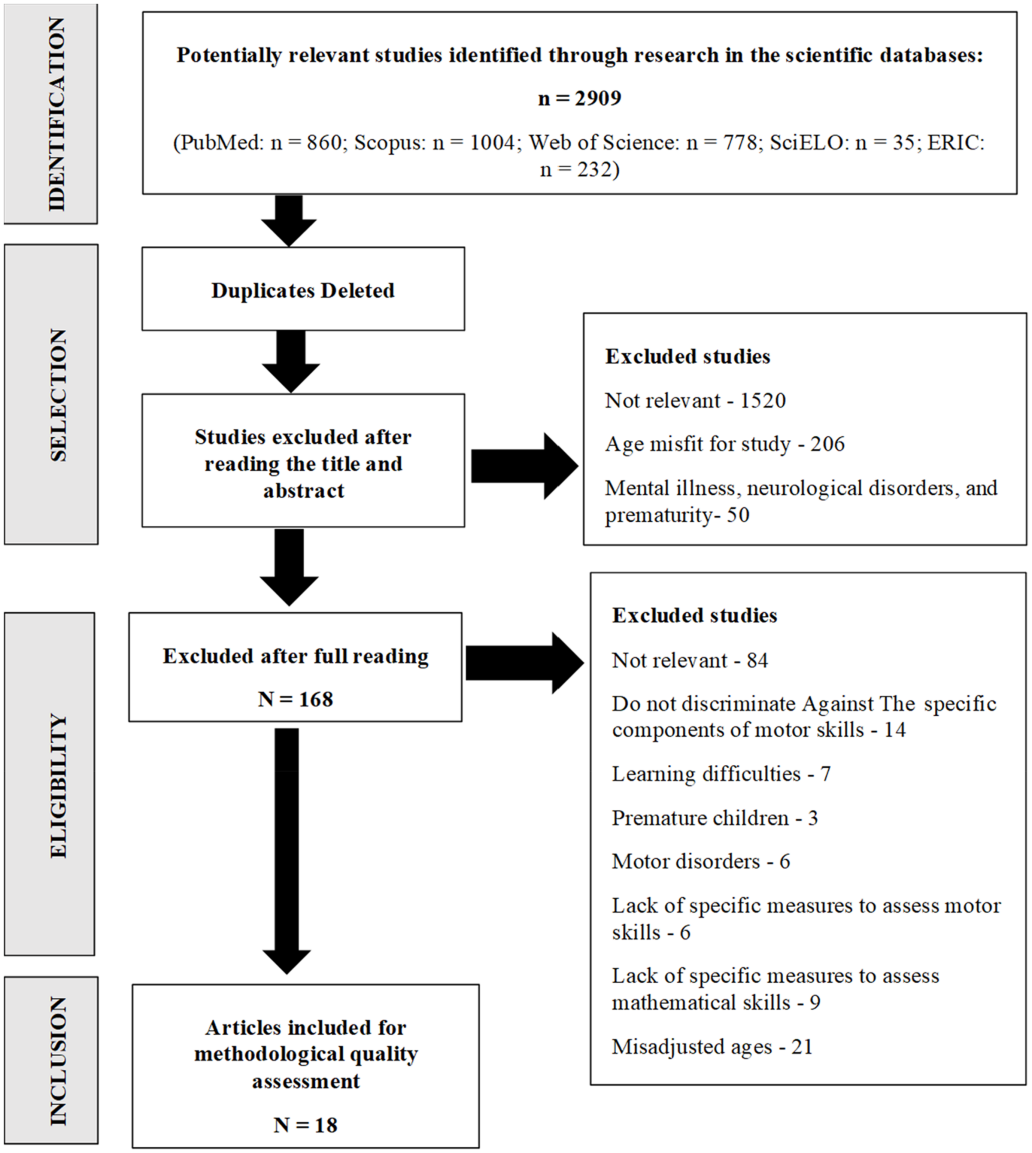
Flowchart of research processes for the inclusion of studies (PRISMA).

The total search result in the databases produced 2,909 studies (PubMed: *n* = 860; Scopus: *n* = 1,004; Web of Science: *n* = 778; SciELO: *n* = 35; ERIC: *n* = 232), of which 965 were automatically excluded because they were duplicates and, after reading the title and abstract, 1,776 studies were excluded, thus resulting in a total of 168 studies potentially suitable studies. After reading these studies in full, 150 were excluded because they did not meet all inclusion criteria and, so, only 18 were included. After selecting the studies included for the review, a manual search of their bibliographic references was performed, from which no study was obtained, either because they did not meet all the eligibility criteria or because they were already included in the selection. The flowchart according to PRISMA methodology is shown in Figure 3.

Of the 18 studies included in this review, the following procedures were performed: Methodological quality assessment (3.2); Analysis of the main characteristics of the studies (3.3); Association between motor skills and the performance of mathematical competences (3.3); Identification of the instruments used in the studies to assess motor skills and mathematical performance (3.4).

### 3.2. Methodological quality assessment

With regard to the studies’ rating, observed through MQCOM, on a scale from 0 to 16 points, the studies with the lowest rating obtained 12 points (Suggate et al., 2017; Fischer et al., 2018, 2020; Clark et al., 2021) and the highest rating was obtained by the study of Escolano-Pérez et al. (2020) (Table 1). Regarding the methodological characteristics, all 18 studies make reference to the methodology used (item 1), use observation units with criteria (item 4), fit the observation instruments to the study (item 8), the data and parameters are specified in the study (items 11 and 12), and all studies indicate having performed some inferential analysis to analyze the data. It is noteworthy that all studies perform a good interpretation of the results in the discussion section (item 16), except for the studies of Brock et al. (2018) and Osorio-Valencia et al. (2017) which partially met this criterion. Among all the studies, only one does not partially meet the theoretical framework (item 4) nor does it specify the coding manual with definition of the categories/behaviors and specification of the dimensions (Osorio-Valencia et al., 2017). The major limitation of the studies was the non-use of software for data collection, since only the study by Escolano-Pérez et al. (2020) met this criterion and the study by Clark et al. (2021) partially met it. Probably the design of the studies did not require the use of specific software for data collection, analysis, and interpretation.

**Table 1:** Methodological characteristics observed using the Methodological Quality Checklist for studies based on Observational Methodology (MQCOM).

Author and year	Item																Total
	Reference to observational methodology	Delimitation of study objectives	Theoretical framework referenced	Observation unit criteria	Temporal criteria	Dimensionality criteria	Inclusion/exclusion criteria	Adequacy of the observation instrument	Coding manual	Software usage	Data type specification	Parameters specification	Session delimitation	Inter-observer reliability	Type of data analysis	Interpretation of results in the discussion	
Clark et al. (2021)	1	0.5	1	1	0	1	0	1	1	0.5	1	1	1	0	1	1	12
Khng and Ng (2021)	1	0.5	1	1	1	1	0.5	1	1	0	1	1	1	0	1	1	13
Escolano-Pérez et al. (2020)	1	1	1	1	1	1	1	1	1	1	1	1	1	1	1	1	16
Fischer et al. (2020)	1	0.5	1	1	0.5	1	0.5	1	1	0	1	1	0.5	0	1	1	12
Greenburg et al. (2020)	1	1	1	1	1	1	1	1	1	0	1	1	1	1	1	1	15
Cameron et al. (2019)	1	1	1	1	1	1	0.5	1	1	0	1	1	1	1	1	1	14.5
de Waal (2019)	1	1	1	1	1	1	1	1	1	0	1	1	1	0	1	1	14
Nesbitt et al. (2019)	1	1	1	1	1	1	1	1	1	0	1	1	1	1	1	1	15
Brock et al. (2018)	1	1	1	1	0.5	0.5	0.5	1	1	0	1	1	0.5	1	1	0.5	12.5
Duran et al. (2018)	1	1	1	1	1	1	1	1	1	0	1	1	1	1	1	1	15
Fischer et al. (2018)	1	1	1	1	0	1	0.5	1	1	0	1	1	0.5	0	1	1	12
Kim et al. (2018)	1	1	1	1	1	1	0.5	1	1	0	1	1	1	1	1	1	14.5
Manfra et al. (2017)	1	1	1	1	1	1	1	1	1	0	1	1	1	1	1	1	15
Osorio-Valencia et al. (2017)	1	0.5	0.67	1	0.5	0.5	1	1	0.5	0	1	1	1	1	1	0.5	12.17
Suggate et al. (2017)	1	0.5	1	1	0.5	1	0.5	1	1	0	1	1	0.5	0	1	1	12
Becker et al. (2014)	1	1	1	1	0.5	1	0.5	1	1	0	1	1	0.5	0	1	1	12.5
Verdine et al. (2014)	1	1	1	1	0.5	1	0.5	1	1	0	1	1	0.5	1	1	1	13.5
Dinehart and Manfra (2013)	1	1	1	1	1	1	1	1	1	0	1	1	1	1	1	1	15
Total	18	15.5	17.7	18	13	17	12.5	18	17.5	1.5	18	18	15	12	18	17	

1 = meets the criteria; 0.5 = partially complies; 0 = does not comply.

### 3.3. Characteristics of studies

The 18 studies included in this review were observational studies, 11 (61%) longitudinal and 7 (39%) transversals, which identified associations between motor skills and mathematical performance, using validated and reliable instruments for this purpose, in typically-developed children of both sexes, who attended preschool education. Regarding their country of origin, the majority of studies, 10 (56%), were conducted in the United States (Dinehart and Manfra, 2013; Verdine et al., 2014; Manfra et al., 2017; Brock et al., 2018; Duran et al., 2018; Kim et al., 2018; Cameron et al., 2019; Nesbitt et al., 2019; Greenburg et al., 2020; Clark et al., 2021), followed by three from Germany (17%; Suggate et al., 2017; Fischer et al., 2018, 2020). The remaining five studies were carried out in different countries, namely Singapore (Khng and Ng, 2021), Spain (Escolano-Pérez et al., 2020), South Africa (De Waal, 2019), Mexico (Osorio-Valencia et al., 2017), and Northeast Pacific (Becker et al., 2014; Table 2).

**Table 2:** Characteristics of studies that investigated the relationship between motor skills and mathematic performance in children who attended the preschool education with typical development.

Author country type of study	*N*	Sex	Age in years (mean)	Aim of the study	Study results and conclusions
Clark et al. (2021) United States Transverse	33	D	4–5 (4.66)	To investigate the association between fractional tasks and performance in fine motor skills and the use of gesture while counting	Performance in the**FMC** significantly predicts fractional reasoning tasks (*R2* = 0.258; *p* = 0.003)
Khng and Ng (2021) Singapore Transverse	1.248	M 614 F 631	(4.78)	Examines the interaction between FMS and executive function in the simultaneous prediction of mathematics, reading and spelling in early kindergarten	**VMI** significantly predicted math performance (*r* = 0.637; *p* < 0.01). Identifying VMI difficulties early in kindergarten may be important for diagnosing learning difficulties in mathematics
Escolano-Pérez et al. (2020) Spain Longitudinal (1 year)	38	M 12 F 16	5–6 (5.72)	Assess in the last year of preschool the specific components of GMS and FMS and 1 year later and link to academic skills (literacy and math)	Of all the specific motor skills (GMS and FMS), only**VMI** predicts later math performance (*β* = 0.476; *p* = 0.002; R2 = 0.227; R2aj = 0.205; *p* = 0.003). Early assessment of VMI is critical to identify academic performance
Fischer et al. (2020) Germany Transverse	80	M 40 F 40	3.1–6.3 (4.80)	Verify that the FMS (FMC and IVM) are associated with finger-based numerical representations (ordinal and cardinal)	Only the**FMC** was related to numerical representations based on the fingers (ordinal: *r* = 0.751; *p* < 0.01; cardinal: *r* = 0.781; *p* < 0.01). Finger counting habits are a predictor of mathematical performance
Greenburg et al. (2020) United States Longitudinal (4 years)	34.491	D	(4.68)	Examine the importance of FMS in preschool for later school performance (3rd, 4th and 5th grade)	Both VSI and FMC were significantly associated with mathematics performance in later years (VSI: 0.15 > *β* > 0.10; *p* < 0.01; FMC: 0.06 > *β* > 0.05; *p* < 0.01).**VSI** and**FMC** are predictors of later school mathematics performance
Cameron et al. (2019) United States Longitudinal (1 year)	555	D	(T1 5.28 T2 6.28)	Examine associations between cognitive and academic skills: executive function, VMI, mathematics, and letter and word knowledge	Children with better**VMI** showed better results in solving applied problems (T1: *r* = 0.48; *p* < 0.001; T2: *r* = 0.48; *p* < 0.001), contributing to early learning of mathematical problems
De Waal (2019) South Africa Transverse	69	M 38 F 31	5–6 (6.1)	Determine the correlation between motor skills and academic performance	Of the GMS,**balance** (dynamic and static) correlated moderately and significantly with math performance (0.46 > *r* > 0.23; *p* < 0.05). Children should be exposed to activities that include balance to improve math performance
Nesbitt et al. (2019) United States Longitudinal (2 years)	1.138	M 620 F 518	(T1 4.5 T4 6.4)	To examine the longitudinal associations between VMI, executive function, and mathematics performance	The increase in math performance over time is a product of the influence of executive functions and**VMI** (T1: *r* = 0.29; *p* < 0.01; T2: *r* = 0.22; *p* < 0.01; T3: *r* = 0.19; *p* < 0.01; T4: *r* = 0.23; *p* < 0.01). Executive functions and VMI positively influence later mathematics performance
Brock et al. (2018) United States Longitudinal (3 years)	256	M 119 F 137	4.8–6.4 (5.41)	To explore the associations between executive function, VMI, and performance in reading and mathematics from kindergarten through second grade in economically disadvantaged children	**VMI** in kindergarten only predicts math performance in grade 1 (T2) (T1 to T2: *r* = 0.17; *p* < 0.05). Early interventions must be performed to develop VMI
Duran et al. (2018) United States Longitudinal (6.5 months)	162	M 81 F 81	(T1 5.5 T2 6.6)	To examine the associations between executive function, VMI, and mathematics performance in kindergarten and later in early first grade in students with low socioeconomic status	**VMI** is related to math performance (VMI – WJ III: *r* = 0.50; *p* < 0.05; VMI – TEMA: *r* = 0.53; *p* < 0.05; VMI – KeyMath3: *r* = 0.53; *p* < 0.05) and appears to have an additional and unique association in improving their performance
Fischer et al. (2018) Germany Transverse	177	M 87 F 90	2.7–6.4 (4.6)	Investigate the relationship between FMC and procedural counting skills as well as conceptual counting knowledge	**FMC** is strongly related to procedural counting skills (FMC – Procedural counting: *r* = 0.41; *p* < 0.01) and (FMC conceptual – Conceptual counting: *r* = 0.36; *p* < 0.01), being a necessary prerequisite for sensorimotor experience of numbers through counting by fingers
Kim et al. (2018) United States Longitudinal (2 years)	134	D	4.9–6.8 (5.6)	Explore the longitudinal associations between VMI, attention, FMC, and math skills	Over time**VMI** predicted changes in math skills (VMI – KeyMath3: T1-T2: *β* = 0.13; < 0.001; T2-T3: *β* = 0.14; *p* < 0.001) and**FMC** was indirectly related as math performance through VMI (FMC – VMI: T1-T2: *β* = 0.18; < 0.01; T2-T3: *β* = 0.14; *p* < 0.05). Reciprocal associations exist between VMI, FMC, attention, and mathematics from early childhood through the early years of schooling
Manfra et al. (2017) United States Longitudinal (4 years)	1.442	M 688 F 754	4 years up to 3rd grade.	To explore the association between preschool academic skills and 3rd grade in children from low-income families	**VMI** and**FMC** in preschool math skills in 3rd grade (VMI: *β* = 0.008; < 0.01; FMC: *β* = 0.012; < 0.001)
Osorio-Valencia et al. (2017) Mexico Longitudinal (2 years)	148	D	3–5	To assess motor development in 3-year-old children and its relationship to their cognitive abilities at age 5	Only children’s**FMC** and**VMI** at age 3 significantly influenced math performance at age 5 (FMC – Mat: *β* = 0.74, *p* = 0.005; VMI – Mat: *β* = 2.11, *p* = 0.001). Early motor assessment and stimulation and help create strategies that facilitate the acquisition of academic knowledge
Suggate et al. (2017) Germany Transverse	81	M 40 F 41	3.3–6.3 (4.9)	Explore whether FMC would correlate with numerical ability	**FMC** is closely related to finger-based numerical skills (*β* = 0.23, *p* = 0.05), allowing early development of numerical skills through counting by fingers
Becker et al. (2014) Northeast Pacific Transverse	127	D	4.4–6.6 (5.6)	Explore the contributions of behavioral self-regulation, two measures of executive function, and VMI on academic performance	**VMI** is significantly associated with math performance in the early years (*r* = 0.59; *p* < 0.05; *β* = 0.13; *p* = 0.045), and may be an indicator to inform teaching strategies in children with math difficulties
Verdine et al. (2014) United States Longitudinal (1 year)	44	M 22 F 22	3.2–4 (3.6) and later 4.3–5.2 (4.76).	Determine the contribution of spatial skills and executive function to early mathematics performance	There is a significant association between**VMI** and math skills in both evaluation moments (VMI – WIAT: *r* = 0.673; *p* < 0.001). VMI is a spatial predictor of math skills
Dinehart and Manfra (2013) United States Longitudinal (3 years)	3.224	M 1515 F 1709	(5.3 and 2 years later)	Examines whether the FMS of economically disadvantaged children predicts later academic performance	Children who performed better on the FMS (FMC and VMI) at the end of preschool were those who performed better in math in 2nd grade (FMC-LAP-D: *β* = 1.75; *p* < 0.001; VMI-LAP-D: *β* = 1.20; *p* < 0.001). FMS in preschool predicts math performance in 2nd grade

D, Does not discriminate; F, female; FMC, fine motor coordination; M, male; Mat, mathematics; *N*, number; *p*, significance level; *r* and *R*^2^, correlation; T, time; TEMA-3, Test of early mathematics ability, 3rd edition; VMI, visuomotor integration; VSI, visuospatial integration; LAP-D, Learning Accomplishment Profile Diagnostic, 3rd Editions; T, assessment moment; WIAT – III, Wechsler Individual Achievement Test, 3rd edition; WJ-III, Woodcock-Johnson Achievement Test.

Regarding the sample size, the studies presented a minimum of 33 (Clark et al., 2021) and a maximum of 34.491 (Greenburg et al., 2020), with a total of 43.447 participants. All studies included children of both sexes. Regarding teaching frequency, most studies, 12 (67%), exclusively included children from preschool education, seven transversals (Becker et al., 2014; Suggate et al., 2017; Fischer et al., 2018; De Waal, 2019; Fischer et al., 2020; Clark et al., 2021; Khng and Ng, 2021) and five longitudinal (Verdine et al., 2014; Osorio-Valencia et al., 2017; Kim et al., 2018; Cameron et al., 2019; Escolano-Pérez et al., 2020). The remaining six studies (33%) were longitudinal and included children from preschool education and later years (Dinehart and Manfra, 2013; Manfra et al., 2017; Brock et al., 2018; Duran et al., 2018; Nesbitt et al., 2019; Greenburg et al., 2020; Table 2). Regarding age, in studies that included only preschool education, children were aged between 2.7 (Fischer et al., 2018) and 6.8 (Kim et al., 2018), and in studies that involved children from preschool education and beyond, the minimum age was 4 years and the maximum was up to the 5th grade (children between 10 and 11 years old; Greenburg et al., 2020).

The study conducted by Greenburg et al. (2020) was included in this review as the authors distinguished in their research two specific aspects of FMS (FMC and the integration of motor information with visual (VMI) and spatial (VSI) information), addressing in this study these specific motor skills only as VSI and not VMI skills (Table 2).

### 3.4. Associations between motor skills and mathematical performance

Regarding the study of the associations between motor skills and mathematics, considering the main conclusions from the 18 studies, only in the study carried out by De Waal (2019) whose objective was to determine if there were correlations between motor skills and the academic performance in preschoolers aged 5–6 years old, there was a moderate and significant correlation between a specific component of GMS, namely balance skills (dynamic and static) and mathematical performance (0.46 > *r* > 0.23; *p* < 0.05). On the other hand, FMS have been reported to be associated with mathematical performance in 17 studies (Table 2).

**Figure 4:**
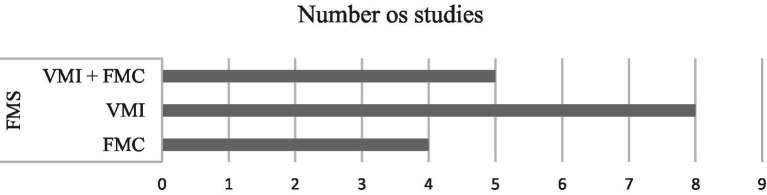
Distribution of studies associating FMS, FMC and VMI to math skills. FMC, fine motor coordination; FMS, fine motor skills; VMI + FMC, visuomotor integration plus fine motor coordination.

When analyzing the distribution of studies that associated mathematical performance with FMS, namely FMC and VMI, it was found that eight studies reported VMI (Becker et al., 2014; Verdine et al., 2014; Brock et al., 2018; Duran et al., 2018; Cameron et al., 2019; Nesbitt et al., 2019; Escolano-Pérez et al., 2020; Khng and Ng, 2021), four studies FMC (Suggate et al., 2017; Fischer et al., 2018, 2020; Clark et al., 2021) and five studies reported both FMS (FMC + VMI; Dinehart and Manfra, 2013; Manfra et al., 2017; Osorio-Valencia et al., 2017; Kim et al., 2018; Greenburg et al., 2020; Figure 4).

Among the VMI, FMS was the one that stood out the most among the 18 studies, having been reported in 13 (72%), and FMC was associated with mathematical performance only in nine (50%). Thus, among FMS, VMI (more than FMC) was more frequently associated with mathematical performance, and is still reported as an important factor for the diagnosis of learning disabilities in mathematics (Khng and Ng, 2021). However, both VMI and FMC were mentioned as strong predictors of mathematical performance in the present (VMI: Dinehart and Manfra, 2013; Becker et al., 2014; Duran et al., 2018; Khng and Ng, 2021; FMC: Dinehart and Manfra, 2013; Suggate et al., 2017; Fischer et al., 2020) and in the future (VMI: Verdine et al., 2014; Manfra et al., 2017; Osorio-Valencia et al., 2017; Brock et al., 2018; Cameron et al., 2019; Escolano-Pérez et al., 2020; Greenburg et al., 2020; CMF: Manfra et al., 2017; Osorio-Valencia et al., 2017; Greenburg et al., 2020).

**Figure 5:**
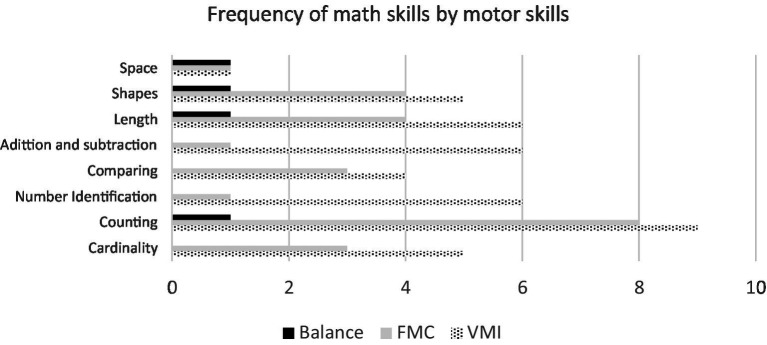
Distribution of studies associating FMS (FMC and VMI) and balance with math skills. FMC, fine motor coordination; VMI, visuomotor integration.

In the frequency analysis of the association between MS and math skills (Figure 5 and Tables 3, 4), regarding GMS, namely balance, it was associated with the following math skills: counting, measurement (length), shapes and spatial relations (De Waal, 2019). Regarding FMS, both FMC and VMI were associated with all math skills (Figure 5; Tables 3, 4). However, counting was the math skill that was most frequently reported to be associated with FMS. It was associated with FMC in all nine studies (100%; Dinehart and Manfra, 2013; Manfra et al., 2017; Osorio-Valencia et al., 2017; Suggate et al., 2017; Fischer et al., 2018; Kim et al., 2018; Fischer et al., 2020; Greenburg et al., 2020; Clark et al., 2021) and with VMI (Dinehart and Manfra, 2013; Verdine et al., 2014; Manfra et al., 2017; Osorio-Valencia et al., 2017; Duran et al., 2018; Kim et al., 2018; Escolano-Pérez et al., 2020; Greenburg et al., 2020; Khng and Ng, 2021) in nine studies (53%). One can highlight the fact that measurement skills (length), shapes (Dinehart and Manfra, 2013; Manfra et al., 2017; Kim et al., 2018; Greenburg et al., 2020), cardinality (Osorio-Valencia et al., 2017; Fischer et al., 2018; Kim et al., 2018; Fischer et al., 2020), and comparing (Osorio-Valencia et al., 2017; Fischer et al., 2018, 2020; Clark et al., 2021) were reported in four studies. Only in one study number identification (Kim et al., 2018), addition, and subtraction (Suggate et al., 2017) were reported to be associated with FMC. Regarding VMI, except the math skill spatial relations, which was only reported in a single study (Kim et al., 2018), all other skills were reported in four or more studies. Thus, in six studies the number identification skill was reported (Verdine et al., 2014; Duran et al., 2018; Kim et al., 2018; Nesbitt et al., 2019; Escolano-Pérez et al., 2020; Khng and Ng, 2021), addition and subtraction (Becker et al., 2014; Brock et al., 2018; Duran et al., 2018; Cameron et al., 2019; Nesbitt et al., 2019; Khng and Ng, 2021), and measurement (length; Dinehart and Manfra, 2013; Manfra et al., 2017; Duran et al., 2018; Kim et al., 2018; Nesbitt et al., 2019; Greenburg et al., 2020). In five studies, the cardinality (Verdine et al., 2014; Osorio-Valencia et al., 2017; Duran et al., 2018; Kim et al., 2018; Escolano-Pérez et al., 2020) and shapes skills (Dinehart and Manfra, 2013; Manfra et al., 2017; Duran et al., 2018; Kim et al., 2018; Greenburg et al., 2020) were reported. The comparing skill was reported in four studies (Verdine et al., 2014; Osorio-Valencia et al., 2017; Nesbitt et al., 2019; Khng and Ng, 2021).

**Table 3:** Frequencies of associations between motor skills and mathematics by teaching frequency.

Author and year	Teaching frequency	Motor Skills			Math Skills							
		FMC	VMI	Bl	A	B	C	D	E	F	G	H
Clark et al. (2021)	Preschool education	**X**				**+**		**+**				
Khng and Ng (2021)	Preschool education		**X**			**+**	**+**	**+**	**+**			
Escolano-Pérez et al. (2020)	Preschool education		**X**		**+**	**+**	**+**					
Fischer et al. (2020)	Preschool education	**X**			**+**	**+**		**+**				
Greenburg et al. (2020)	From Preschool education to 5th grade	**X**	**X**			**+**				**+**	**+**	
Cameron et al. (2019)	Preschool education		**X**						**+**			
De Waal (2019)	Preschool education			**X**		**+**				**+**	**+**	**+**
Nesbitt et al. (2019)	Preschool education		**X**				**+**	**+**	**+**	**+**		
Brock et al. (2018)	Preschool education to 2nd grade		**X**						**+**			
Duran et al. (2018)	Preschool education to 1st grade		**X**		**+**	**+**	**+**		**+**	**+**	**+**	
Fischer et al. (2018)	Preschool education	**X**			**+**	**+**		**+**				
Kim et al. (2018)	Preschool education	**X**	**X**		**+**	**+**	**+**			**+**	**+**	**+**
Manfra et al. (2017)	Preschool education to 3rd grade	**X**	**X**			**+**				**+**	**+**	
Osorio-Valencia et al. (2017)	Preschool education	**X**	**X**		**+**	**+**		**+**				
Suggate et al. (2017)	Preschool education	**X**				**+**			**+**			
Becker et al. (2014)	Preschool education		**X**						**+**			
Verdine et al. (2014)	Preschool education		**X**		**+**	**+**	**+**	**+**				
Dinehart and Manfra (2013)	Preschool education to 3rd grade	**X**	**X**			**+**				**+**	**+**	
TOTAL		9	13	1	7	14	6	7	7	7	6	2

Math Skills: (A – cardinality; B – Counting; C – Numbers Identification; D – Comparing; E – Addition and subtraction; F – Length; G – Shapes; H – Space); FMC, Fine Motor Coordination; MS, Motor Skills; Bl, Balance; VMI, Visuomotor Integration.

**Table 4:** Instruments used in the association between motor skills and math skills.

Author (year)	Motor skills			Math skills	
	Instr	MS	Evaluation	Instruments	Skills
Clark et al. (2021)	GPT	FMC	The GPT task was used to fit pins into hole. It is a widely used measure that requires dexterity and manual speed	Shared story task	Counting and comparing
				Point counting task	
Khng and Ng (2021)	IED III	VMI	Use of the Fine Motor Subscale. The tests administered were: Visual motor skills; Drawing of a person; Writing the sequence of numbers; Sequential drawing of capital letters	TEMA-3 (Formal and informal knowledge)	Counting, comparing, numbers identification, addition and subtraction
Escolano-Pérez et al. (2020)	VMI	VMI	The VMI was assessed using the tests: Copy Shapes, Letters Words and Numbers	PAIB-1 (Basic Aspects of Mathematics Quiz)	Cardinality, counting and identification of numbers
Fischer et al. (2020)	MABC-2	FMC	The FMC was evaluated using the Manual Dexterity Scale: placing coins in a box with a slot; string beads on a cord	Finger-Based Number Representations (finger counting; finger montring)	Counting and cardinality (Finger-Based Number Representations); Comparing and counting (Numerical tasks)
				Numerical Tasks (Non-symbolic dot comparison; Symbolic number comparison; Verbal counting sequence)	
Greenburg et al. (2020)	LAP-D	VSI	The VSI was assessed by the Writing Subscale, which includes tasks with pencil and paper, such as copying numbers, letters, and shapes, and drawing objects. The FMC was evaluated by the object manipulation subscale with paper folding, building blocks, cutting with scissors	LAP-D (Counting and matching subscales)	Counting, measurement (length) and shapes
		FMC			
Cameron et al. (2019)	VMI	VMI	The VMI was evaluated through the test VMI that assesses the visual and motor skills in an integrated way. The test requires the child to copy increasingly complex geometric figures as the test progresses	WJ-III (Application of problems subscale)	Addition and subtraction
De Waal (2019)	Kinder kinetics Screening	Balance	Assesses basic movement skills, locomotor, postural (static and dynamic balance) and manipulative in children aged 3–6 years	Foundations for learning: Grade R Assessment Framework R	Counting, measurement (length), shapes and space
Nesbitt et al. (2019)	CDT	VMI	The VMI was evaluated by the task of copying geometric drawings	WJ-III (Application of problems sub-scale and quantitative concepts sub-scale)	Addition and subtraction (application of problems sub-scale); numbers identification; Comparing and measurement (length) (quantitative concepts sub-scale)
Brock et al. (2018)	VMI	VMI	Idem Cameron et al. (2019)	WJ-III (Application of problems subscale)	Addition and subtraction
Duran et al. (2018)	NEPSY	VMI	The VMI was evaluated by the NEPSY Design Copying Subtest. In this test, the children used paper and pencils to copy two-dimensional geometric drawings of increasing complexity	WJ-III (Application problems subscale)	Addition and subtraction (WJ-III); Number identification, shapes and measurement (length) (KeyMath3); Counting, cardinality, number identification, adding and subtracting and measurement (length) (TEMA-3)
				KeyMath-3 (Numeracy, geometry and measurement subscales)	
				TEMA-3 (Formal and informal concepts and skills)	
Fischer et al. (2018)	BEFMF	FMC	3 tasks: Pegboard task (inserting pins into a board), Bead threading (threading beads) and Block turning (turning cylinders, measured speed and fine motor coordination). These are measures used that require manual dexterity	TEDI-MATH (Counting subtest)	Counting, cardinality and comparing
Kim et al. (2018)	NEPSY	VMI	The VMI was evaluated by the Design Copying subtest. In this test, the children used paper and pencils to copy two-dimensional geometric drawings of increasing complexity	KeyMath-3 (Numeracy, geometry and measurement subscales)	Cardinality, counting, number identification, shapes, space and measurement (length)
		FMC	The FMC was evaluated by the *precision* subtest and assesses the speed and accuracy of hand-eye coordination. The total score considers the speed and accuracy scores		
Manfra et al. (2017)	LAP-D	FMC	The FMC was assessed by the Fine Motor Manipulation Subscale (manipulating small objects)	LAP-D (Count and correspondence subscales)	Counting, measurement (length) and shapes
		VMI	The VMI was assessed by the Writing Subscale which includes tasks with pencil and paper, such as copying numbers, letters, and shapes and drawing objects		
Osorio-Valencia et al. (2017)	PDMS-2	VMI	VMI was evaluated through the prehension and visuomotor integration subtests	MSCA (Quantitative scale)	Cardinality, counting and comparing
Suggate et al. (2017)	BEFMS	FMC	3 tasks: Pegboard task (inserting pins into a board), Bead threading (threading beads) and Block turning (turning cylinders, measured speed and fine motor coordination). These are measures used that require manual dexterity	Numerical skills	Counting and adding and subtracting
				Nonfinger-based numerical skills	
				Finger-based numerical skills	
Becker et al. (2014)	VMI	VMI	The VMI was assessed using the VMI which evaluates visual and motor skills in an integrated manner. The test requires the child to copy increasingly complex geometric figures as the test progresses	WJ – III (Application of problems subscale)	Addition and subtraction
Verdine et al. (2014)	VMI	VMI	The VMI was assessed using the VMI which evaluates visual and motor skills in an integrated manner. The test requires the child to copy increasingly complex geometric figures as the test progresses	WIAT-III (Problem-solving subtest)	Counting, cardinality, number identification and comparing
Dinehart and Manfra (2013)	LAP-D	FMC	The FMC was assessed by the Fine Motor Manipulation Subscale (manipulating small objects)	LAP-D (Counting and matching subscales)	Counting, measurement (length) and shapes
		VMI	The VMI was assessed by the Writing Subscale which includes tasks with pencil and paper, such as copying numbers, letters, and shapes and drawing objects		

BEFMS, Battery designed to provide an estimate of children’s fine motor skills in preschool; FMC, Fine Motor Coordination; MS, Motor Skills; IED III, The Brigance Inventory of Early Development III; Instr, Instruments; VMI, visuomotor integration; KeyMath3, KeyMath-3 Diagnostic assessment; LAP-D, Learning Accomplishment Profile-Diagnostic, 3rd edition; MABC-2, Movement Assessment Battery for Children, 2nd edition; PDMS, Peabody developmental motor scale, 2nd edition; MSCA, McCarthy Scales of Children’s Abilities; NEPSY, NEuroPSYchological assessment battery; PAIB-1, Test of basic instrumental aspects: Reading, writing and numeric concepts; CDT, Copy Design Task; TEDI-MATH, Test for the diagnosis of basic skills in mathematics; TEMA-3, Test of Early Mathematics Ability – 3rd edition; GPT, Grooved Pegboard Test; VMI, Test of Visual-Motor Integration; WIAT-III, Wechsler Individual Achievement Test, 3rd edition; WJ-III, Woodcock-Johnson Tests of Achievement.

When analyzing data from studies exclusively involving children who attended preschool, it was found that all math skills were associated with both FMC and VMI: counting – in six studies – (Osorio-Valencia et al., 2017; Suggate et al., 2017; Fischer et al., 2018; Kim et al., 2018; Fischer et al., 2020; Clark et al., 2021) and cardinality (Osorio-Valencia et al., 2017; Fischer et al., 2018; Kim et al., 2018; Fischer et al., 2020) and comparing – (Osorio-Valencia et al., 2017; Fischer et al., 2018, 2020; Clark et al., 2021) in four studies – were the most frequently associated with FMC. Regarding VMI, the math skills most frequently associated with it were counting (Verdine et al., 2014; Osorio-Valencia et al., 2017; Kim et al., 2018; Escolano-Pérez et al., 2020; Khng and Ng, 2021) and number identification (Verdine et al., 2014; Kim et al., 2018; Nesbitt et al., 2019; Escolano-Pérez et al., 2020; Khng and Ng, 2021), both in five studies, and cardinality (Verdine et al., 2014; Osorio-Valencia et al., 2017; Kim et al., 2018; Escolano-Pérez et al., 2020), comparing (Verdine et al., 2014; Osorio-Valencia et al., 2017; Nesbitt et al., 2019; Khng and Ng, 2021), and addition and subtraction (Becker et al., 2014; Cameron et al., 2019; Nesbitt et al., 2019; Khng and Ng, 2021) in four studies.

These results suggest that counting, cardinality, and comparing were the math skills most associated with FMC; and counting, number identification, cardinality, comparing, and addition and subtraction with VMI. However, the math skill of counting was the most commonly associated with FMS (FMC and VMI), as of the 17 studies that analyzed this math skill, it was associated with FMS in 13 (76%; Tables 3, 4).

### 3.5. Instruments used in the studies to assess motor and math skills

In the 18 studies included, 10 instruments were used to assess motor skills (Table 4).

In the only study that associated GMS with mathematical performance (De Waal, 2019), the Kinder kinetics Screening test was used (Pienaar et al., 2016).

To assess FMS, nine instruments were used. To exclusively assess FMC, three instruments were identified: Grooved Pegboard Test (GPT, Strauss et al., 2006; Clark et al., 2021); Movement Assessment Battery for Children, 2nd edition (MABC-2, Henderson et al., 2007; Fischer et al., 2020); Battery designed to provide an estimate of children’s fine motor skills in preschool (BEFMS, Martzog, 2015). To exclusively assess VMI, four instruments were identified: The Brigance Inventory of Early Development III — Standardized (IED III, French, 2013; Khng and Ng, 2021); Test of Visual-Motor Integration, 6th edition (VMI, Beery and Beery, 2010; Becker et al., 2014; Verdine et al., 2014; Brock et al., 2018; Cameron et al., 2019; Escolano-Pérez et al., 2020); Copy Design Task (CDT, Osborn et al., 1984; Nesbitt et al., 2019); NEuroPSYchological assessment battery, 2nd edition (NEPSY, Korkman et al., 1998; Duran et al., 2018). To simultaneously assess FMC and VMI, three instruments were identified: Learning Accomplishment Profile-Diagnostic, 3rd edition (LAP-D, Nehring et al., 1992; Dinehart and Manfra, 2013; Manfra et al., 2017; Greenburg et al., 2020); (NEPSY, Korkman et al., 1998; Kim et al., 2018); Peabody developmental motor scale, 2nd edition (PDMS-2, Folio and Fewell, 2000; Osorio-Valencia et al., 2017; Table 4).

For the assessment of mathematical performance, 16 instruments were identified among the 18 studies (Table 4).

Only in one study it was identified: Mathematics Sharing Stories, Cwikla, 2014 (Clark et al., 2021); Dot Counting Task, Clark et al., 2021 (Clark et al., 2021); Test of basic instrumental aspects: reading, writing and numerical concepts (PAIB-1, Galve-Manzano et al., 2009; Escolano-Pérez et al., 2020); Finger-based number representations (Wasner et al., 2015; Fischer et al., 2020); Number tasks (Nosworthy, 2013; Fischer et al., 2020); Foundations for learning: Assessment Framework Grade R (De Waal, 2019); McCarthy Scales of Children’s Abilities (MSCA, McCarthy, 2004; Osorio-Valencia et al., 2017); Numerical skills (Dowker, 2008; Suggate et al., 2017); Nonfinger-based numerical skills and Finger-based numerical skills (Crollen et al., 2011; Suggate et al., 2017); Wechsler Individual Achievement Test, 3rd edition (WIAT-III, Wechsler, 2007; Verdine et al., 2014).

It was identified in two studies: Test of Early Mathematics Ability – 3rd edition (TEMA-3, Ginsburg and Baroody, 2003; Duran et al., 2018; Khng and Ng, 2021); Test for diagnosing basic math skills (TEDI-MATH, Kaufmann et al., 2009; Fischer et al., 2018, 2020); KeyMath-3 Diagnostic assessment (KeyMath3, Connolly, 2007; Duran et al., 2018; Kim et al., 2018).

The Learning Accomplishment Profile-Diagnostic, 3rd edition (LAP-D, Nehring et al., 1992; Dinehart and Manfra, 2013; Manfra et al., 2017; Greenburg et al., 2020) was identified in three studies.

The Woodcock-Johnson Tests of Achievement (WJ-III, Woodcock et al., 2001; Becker et al., 2014; Brock et al., 2018; Duran et al., 2018; Cameron et al., 2019; Nesbitt et al., 2019) was identified in five studies.

Thus, of the seven instruments used to assess VMI, the VMI test (Beery and Beery, 2010) was the most used, as it was reported in five studies. To assess FMC, the most commonly used instrument was the LAP-D (Nehring et al., 1992) in three studies (Table 4).

The main characteristics of the instruments used in the assessment of VMI require tasks of copying geometric figures, letters, numbers or objects, using a sheet of paper and pencil. To assess FMC, the main characteristics of the instruments used allowed the assessment of dexterity and manual speed through tasks that demand the handling and manipulation of objects such as threading beads, placing coins, building blocks, turning cylinders, folding paper or cutting it with scissors.

## 4. Discussion

The objective of this systematic review was to analyze preschool education children with typical development in the association between motor skills and mathematical performance, identify the math skills involved in this association, as well as the instruments used for this purpose to evaluate both motor skills and mathematics.

Based on the results, in relation to the first objective, there was sufficient evidence to support the associations between FMS, namely FMC and VMI, and mathematical academic performance of the children who attended preschool education with typical development. It is noteworthy that only one study has considered associations between a component of GMS, namely balance, and mathematical academic performance (De Waal, 2019).

Similar results to this study were obtained in a systematic review that sought to report the relationships between motor proficiency and academic performance in mathematics and reading in school-age children and adolescents (Macdonald et al., 2018). The authors concluded that FMS were significantly associated with the performance of math skills, particularly in the early school years, and there was no consistency or sufficient evidence to support associations between the specific components of GMS and academic math performance. Likewise, in a systematic review carried out by Van der Fels et al. (2015), in children aged between 4 and 16 years with typical development, most observational studies (86%) reported significant positive associations between FMS and academic performance, especially in children who attended the preschool education up to the 2nd grade.

Studies have shown that GMS are critical for the development of social skills as well as for physical well-being (Cameron et al., 2016), as they influence children’s level of physical activity and health (Logan et al., 2015; Hamilton and Liu, 2018). On the other hand, FMS have been more strongly associated with academic performance (Cameron et al., 2016) and found to influence children’s ability to perform visual motor integration activities, which is important for classroom activities (Strooband et al., 2020). It has been suggested that, at school age, GMS may be important for social affirmation (Ommundsen et al., 2010) and perceived athletic competence (Piek et al., 2006), and FMS for school readiness (Grissmer et al., 2010) and perceived academic competence (Piek et al., 2006).

Despite different purposes, research has shown that during child development, GMS and FMS seem to have some correlation (Roebers and Kauer, 2009; Cameron et al., 2012; Dayem et al., 2015; Oberer et al., 2017), as higher order neuromotor processes seem to be involved simultaneously in the learning of GMS and FMS (Roebers and Kauer, 2009; Oberer et al., 2017). However, studies that investigated the relationship between GMS and FMS, at different stages of school education, obtained controversial results when comparing measures of individual performance of GMS and FMS (Cameron et al., 2012; Amaro et al., 2014; Dayem et al., 2015; Tortella et al., 2016; Oberer et al., 2017). Cameron et al. (2012) and Oberer et al. (2017) showed a moderate correlation between GMS and FMS. Specifically, Oberer et al. (2017) reported a positive correlation in children aged between 5.6 and 7.25 years, assessing gross and fine motor skills through speed and accuracy tasks. Likewise, the investigation by Cameron et al. (2012) also reported a positive correlation in younger children (3–4 years) between GMS, namely balance and jumping and jumping tasks, and FMS, namely building tasks using blocks and drawing tasks. In addition, Dayem et al. (2015), showed an even greater correlation between GMS (assessed by locomotor tasks, object manipulation, and balance tasks) and FMS (assessed by writing tasks) in children aged 4–6 years. On the other hand, other authors disagree on the positive correlation between GMS and FMS (Souza et al., 2010; Amaro et al., 2014; Tortella et al., 2016). The study carried out by Tortella et al. (2016) reported that there was no correlation between GMS and FMS in preschool education children aged between 5 and 6 years, evaluating GMS through precision, balance, throwing, and walking tasks, while FMS were evaluated using speed and precision tasks, such as block constructions and placing coins. Additionally, Souza et al. (2010), when investigating global motor performance using the Bayley Scales of Infant and Toddler Development (Third Edition), found a weak correlation between GMS and FMS. Furthermore, Amaro et al. (2014) did not reported any correlation between GMS and FMS in children aged between 5 and 10 years when comparing the scores obtained in the “Körperkoordinationtest für kinder” and the Minnesota manual dexterity test. These contrasting results can be attributed to the fact that motor skills do not follow linear development trajectories (Souza et al., 2010; Flatters et al., 2014a). Therefore, it is not surprising that investigating children of different ages can produce different results. In addition, these studies assessed motor skills over short periods using heterogeneous tasks (Sorgente et al., 2021).

However, it is important to note that in the same action it is complex to unequivocally differentiate the involvement of each of the motor skills (GMS and FMS), as they coexist and are fundamental for the efficient performance of the task, as both are related and influence each other (Payne and Isaacs, 2012; Flatters et al., 2014b). In this sense, GMS are influenced and influence FMS, the first being also a good predictor of children’s school learning (Sortor and Kulp, 2003; Beery and Beery, 2006; Henderson et al., 2007; Spanaki et al., 2008; Kambas et al., 2010; Kadkol et al., 2014; Byers et al., 2016; Africa and Deventer, 2017; Bellocchi et al., 2017).

However, there are very few studies in the literature that investigated the association between GMS and academic performance (De Waal, 2019; Sorgente et al., 2021), so it is a topic that can be the subject of further research.

Although in this review only one study associates GMS, namely balance, with mathematical performance (De Waal, 2019), it should also be analyzed. In a study carried out by Vuijk et al. (2011), the authors also found significant correlations between balance and mathematics, however this study was developed in children with learning difficulties at school age. Balance, especially when vision is not used, depends a lot on the effective functioning of the vestibular system involved in the execution of controlled movements (Cheatum and Hammond, 2000). In this sense, problems in the vestibular system not only lead to delays in motor proficiency, but can also negatively affect the performance of activities in the classroom (Cheatum and Hammond, 2000). However, in a recent systematic review, the results concluded that balance did not significantly correlate with mathematical academic performance, as in other GMS considered (Macdonald et al., 2018). Regarding FMS, of the 17 studies that associated these skills with mathematical performance, VMI was the one that stood out the most, being reported in 13 studies (76%), followed by FMC in nine (53%). However, by itself, this result does not mean that the VMI of FMS will be the most predictive of mathematical performance, as studies that included the two FMS concluded that FMC and VMI were both predictors of mathematical performance (Dinehart and Manfra, 2013; Manfra et al., 2017; Kim et al., 2018; Greenburg et al., 2020), except the study carried out by Fischer et al. (2020), in which FMC was the only one associated with mathematical performance and the studies conducted by Escolano-Pérez et al. (2020) and Osorio-Valencia et al. (2017), in which on the contrary, VMI was the only one associated with mathematical performance. Studies in which only FMC (Suggate et al., 2017; Fischer et al., 2018; Clark et al., 2021) or VMI (Becker et al., 2014; Verdine et al., 2014; Brock et al., 2018; Duran et al., 2018; Cameron et al., 2019; Nesbitt et al., 2019; Khng and Ng, 2021) were analyzed concluded that these motor skills were associated with mathematical performance. Thus, each FMS was used according to the aim of each study. In this sense, although VMI appears as the most frequently associated with mathematical performance, it does not mean *a priori* that it will have a greater degree of importance than FMC. These results suggest that both FMS (FMC and VMI) are important predictors of mathematical performance depending only on the objectives of the studies and instruments used for this purpose.

VMI involves the integration of visual and motor skills (Sortor and Kulp, 2003; Beery and Beery, 2006) coordinated through the fingers and hands, that is, FMC (Gabbard et al., 2001; Beery and Beery, 2006; Feder and Majnemer, 2007; Bezrukikh and Terebova, 2009; Kambas et al., 2010; Kadkol et al., 2014; Byers et al., 2016). In this sense, VMI implies the mental representation of an image that is replicated by controlling the meticulous movement of the fingers (Carlson et al., 2013). Thus, FMC plays a very important role in school success (Sortor and Kulp, 2003; Roebers et al., 2014; Kim et al., 2015; Fischer et al., 2020) because children with better FMC will be better at handling objects such as pencils or notebooks, which allows them to direct additional attention resources toward learning, rather than focusing them on movements associated with FMC (Kim et al., 2018). FMC can also serve as a fundamental competency by which more complex processes can be built (Sortor and Kulp, 2003), namely for the development of VMI skills (Kim et al., 2018). There is also some evidence that FMC is linked to mathematics through its contribution to the development of VMI (Kim et al., 2018). Thus, a child with good FMC, when performing an academic task, may impose a lower cognitive load compared to a child who still has difficulties in FMC (Luo et al., 2007; Cameron et al., 2015). Cameron et al. (2012) conducted an observational study in classrooms at preschool education and found that 46% of the school day was dedicated to activities involving FMS (FMC and VMI). These activities included tasks such as writing, drawing, using scissors to cut paper, bean counting tasks, and playing with toys like building blocks and Legos. In this sense, FMC and VMI are considered essential for early (Lillard, 2005) and interdependent learning (Kim et al., 2018), proving to be a powerful predictor for school readiness (Bala et al., 2010; Grissmer et al., 2010), adaptation and transition from the preschool education to the 1st grade (Bart et al., 2007) and end of 6th grade (Pagani et al., 2010) and also a great predictor of subsequent academic performance, especially in reading and mathematics (Cameron et al., 2012; Dinehart and Manfra, 2013). On the other hand, low levels of performance in FMS are associated with learning difficulties in the areas of reading, writing, and mathematics (Coetzee and Gerber, 2018).

Another objective of this study was to verify the frequencies with which math skills were associated with MS. Regarding GMS, only balance (static and dynamic) was associated with the math skills of counting, measurement (length), shapes, and space (De Waal, 2019). Despite these data, and as only the study of De Waal (2019) with a relatively small sample (*n* = 30) associated balance with math skills, it would be too premature to conclude in a sustained way the influence of this HMG on math skills. It is suggested that further studies consider the association between balance and math skills in order to support such conclusions.

Regarding FMS, all math skills proposed for Preschool Education (National Research Council, 2009) were associated with both FMC and VMI. Regardless of the type and characteristics of the studies, counting was the math skill most often associated with FMS, FMC, and VMI.

Counting can be seen as an infinitely long and ordered list of numbers that allows you to quantify what you want. In essence, counting is a way of doing a 1 to 1 correspondence between objects. Generally, each successive counting number describes a quantity that is one more than the previous quantity. Similarly, counting backwards is subtracting one from the previous number (National Research Council, 2009). In this sense, children, in addition to developing the notion of cardinality (Hannula, 2005), are performing addition and subtraction actions that are fundamental for solving problems (Siegler and Shrager, 1984). Children who make frequent counting errors have difficulty calculating (Geary et al., 2007). Since children show a spontaneous tendency to count, educators should take advantage of this to stimulate their practice with the aim of developing not only counting skills but other associated math skills (Hannula, 2005).

Another objective of this review was to describe the instruments used in the studies to assess and associated motor skills with mathematics. Regarding motor skills, in the 18 studies, 10 instruments were used. The study that assessed and associated one of the GMS (balance) with mathematical skills (De Waal, 2019), the Kinder kinetics Screening test was the instrument used (Pienaar et al., 2016). This instrument assesses the basic skills of fundamental movements in children aged between 3 and 6 years. The skills assessed were the fundamental locomotor, balance (dynamic and static), and object manipulation skills (Pienaar et al., 2016).

Regarding FMS, nine instruments were used in the 17 studies analyzed, three to assess FMC exclusively, four to assess VMI and three to assess both FMC and VMI.

In the assessment of FMS, although the LAP-D (Nehring et al., 1992) was the most used (three studies), its characteristics are similar to the other instruments used only in a study each, namely the NEPSY (Korkman et al., 1998), and the MABC-2 (Henderson et al., 2007), the BEFMS (Martzog, 2015), and the PDMS-2 (Folio and Fewell, 2000). Thus, these instruments assess manual dexterity and speed through tasks such as fitting pins, turning cylinders, stringing beads, building with blocks, folding paper, cutting with scissors, and putting coins in a slot.

In the assessment of VMI, the most commonly used instrument was the VMI (five studies; Beery and Beery, 2010). Although this instrument was the most commonly used, its tasks are also similar to the other instruments used in the assessment of VMI. The main tasks assessed by these instruments consist of copying geometric figures, letters or numbers, and drawing objects using a sheet of paper and pencil.

The literature has shown that the tasks of copying figures or drawing are the most used tasks to assess VMI, and object handling tasks with pinch-like movements are the most used to assess FMC (Davis and Matthews, 2010; Carlson et al., 2013; Newman and Feinberg, 2015; MacDonald et al., 2016).

The literature shows the importance that the instruments used to assess the development of motor skills have in tracking possible difficulties associated with mathematical performance (Kim et al., 2015). Most of these studies used direct neuropsychological assessments (Kim et al., 2015), such as the NEuroPSYchological Assessment Battery – NEPSY (Korkman et al., 1998) and the Visuomotor Integration test (VMI; Beery and Beery, 2010). However, it is recognized that these instruments are usually time consuming and expensive and are only administered by experts for this purpose (Cameron et al., 2012; Williford et al., 2013). In this sense, the characteristics of the instruments most used for the diagnosis of difficulties associated with mathematical performance may be a limitation in relation to the reality of kindergartens. Thus, the main instruments cited in the literature for the assessment of motor skills associated with mathematical performance practically make it impossible for educators to carry out these assessments on their students (Kim et al., 2015).

Regarding the instruments to assess math skills, in the 18 studies analyzed, 16 different instruments were used. Most studies, 78% (14), used only one instrument, the remaining 22% (4) used more than one, depending on the proposed objective. However, the instrument most used to assess math skills was WJ-III (application problems subscale; Woodcock et al., 2001). The WJ-III battery is considered the most complete to explain intellectual functioning, existing in two versions, the first design to assess cognitive abilities (standard form) and the second to assess academic performance (Mather and Gregg, 2002).

It is noteworthy that in the three studies that used the same instrument, namely the LAP-D, to assess FMS (FMC and VMI) and math skills (Dinehart and Manfra, 2013; Manfra et al., 2017; Greenburg et al., 2020) the result was similar, as FMS were associated with the same math skills: counting, measurement (length), and shapes. The LAP-D is an instrument used to measure children’s academic readiness during preschool education. It includes domains for cognitive development, mathematics, and language, and FMS, which includes tasks to assess FMC and VMI (Nehring et al., 1992).

Similarly, in the three studies that used the same instruments for the assessment of both FMS and math skills, the results were similar, i.e., the studies that used the VMI test (Beery and Beery, 2010) for the assessment of FMS and the WJ-III (Woodcock et al., 2001) for the assessment of math skills recorded a significant association between VMI and the same math skills: addition and subtraction (Becker et al., 2014; Brock et al., 2018; Duran et al., 2018; Cameron et al., 2019).

On the other hand, when different instruments were used in the assessment of motor and math skills, the results were different. This suggests that each instrument should measure exactly what it sets out to measure considering the objectives outlined by the researchers (Roberts and Priest, 2006; Mokkink et al., 2010).

The results of this review may have important implications for the implementation of new strategies for teaching mathematics in preschool, as evidence was sufficient to support the influences of FMS (FMC and VMI) on mathematical academic achievement.

It has also been suggested that math skills are improved primarily from an age-adjusted math teaching intervention, better preparing children for school tasks (Sarama and Clements, 2004). However, cognitive skills arise from experiences, motor skills, and sensory-motor skills (Barsalou, 2010; Fischer, 2012). Most often children learn number skills with the help of objects that are typically small (cubes, buttons, etc.; Sarama and Clements, 2016) which requires precise fine motor handling (Luo et al., 2007). This handling will only be effective if the child is aware of the quantities represented by the object (Sarama and Clements, 2016). If the child has a deficit in FMS, it will be more difficult for the child to assign quantitative meaning to objects because the child will focus more on the fine motor actions than on the quantity of the objects (Carr and Davis, 2001). This finding contributes to support that children with learning disabilities in mathematics exhibit the least proficient FMS (Pieters et al., 2012). Since FMS can be automated (Floyer-Lea and Matthews, 2004), interventions in FMS will be recommended to free up cognitive resources for other learning tasks.

In this sense, the preschool curriculum should include guidelines to promote the development of FMS and thus better prepare children for mathematical learning. But, in order to plan a program to promote the development of FMS, a prior assessment of the child is necessary. However, educators lack training to assess FMS, and thus limitations to design appropriate programs adjusted to the needs (Gehris et al., 2015; Cueto et al., 2017).

## 5. Conclusion

This systematic review contributes considerably to the literature, as it found only evidence that supports positive associations between FMS, namely FMC and VMI, and mathematical performance in children with typical development in preschool education, with counting being the mathematical skill most associated with FMS (FMC and VMI). The main characteristics of the instruments used showed that the tasks of copying of figures or drawing are the most used to assess VMI and the tasks of handling objects with pinch-like movements are the most used to evaluate FMC. However, it has been recognized that these instruments are usually time consuming and expensive and are generally only administered by experts for this purpose.

Given the importance of FMS in mathematical skills, there is an urgent need to empower educators with tools to enhance the development of FMS in the classroom context. In this sense, early identification of children with difficulties in fine motor skills will help educators to design better strategies for teaching mathematical skills. Since the initial assessment is fundamental to plan an intervention adjusted to the child, it will be necessary to identify instruments with characteristics that allow their application in the classroom context, i.e., that require little administration time, do not require much experience or training, the possibility of being applied to the group/class, few material resources, and the results can be easily interpreted, classified, and associated with mathematical performance.

### 5.1. Limitations

It is essential to recognize that there were also some limitations in this review. First, it is important to point out that there is an evident gap in the literature of studies that report the association between gross motor skills and mathematical performance in preschool children. Second, there is the considerable heterogeneity of instruments used across studies to assess motor and math skills, making it difficult to compare and clearly interpret the results across studies. Third, the fact that some included studies report covariates that may influence the results, such as demographic factors (for example, socioeconomic status) and cognitive factors (such as executive function and its components). Covariates reported by each eligible study were not discussed as they were beyond the scope of this review.

### 5.2. Recommendations and implications for future research

In order to allow a more accurate comparison of results between studies in the future, researchers should consider consistent use of valid, reliable, and homogeneous standardized instruments. In addition, studies should control demographic, cognitive and physical confounding factors. Finally, as students with neurodevelopmental disorders attend regular schools, future investigations should also examine the relationships between motor and math skills in this population to inform possible forms of intervention.

### 5.3. Recommendations and implications for policies and practices

As it is known that children with FMS difficulties may present negative mathematical performance and given the importance of math performance in future school results, early identification of these difficulties will help educators to design better strategies for teaching math skills. However, although the main instruments reported in the literature accurately assess FMS, these instruments tend to be expensive, time-consuming to administer, require individualized assessments, demand a lot of training and experience, and are usually administered by experts. Thus, the characteristics of most instruments for the evaluation of FMS reveal limitations in the view of our kindergartens’ reality. In this way, their administration in the classroom context by educators can be very conditioned taking into account the characteristics of most classes, which are very numerous and with very extensive curricula.

Due to its importance for academic success, it is considered that educators should carry out an assessment of the development of FMS to their students, in order to detect possible problems associated with mathematical performance. Eventually, if the child shows development problems in FMS, he/she should be referred for a specialized evaluation by a technician with qualifications for this purpose. For this reason, there is an urgent need for a new instrument to evaluate FMS in preschool education children with the ability to adjust to the reality of our kindergartens, that is, one that requires less administration time, does not require much experience or specialized training, has the possibility of being administered to the group/class in a classroom school context, requires few material resources and produces results that can be easily interpreted, classified and associated with mathematical performance. In this sense, this instrument can be a starting point for the early detection of FMS deficits and, consequently, the referral of the child for a new reassessment by a qualified professional. Furthermore, if the child has problems at this level, he or she may benefit from a timely intervention by a specialist and, consequently, prevent/reduce his/her difficulties in mathematical performance.

However, despite FMS being significantly associated with math performance, these skills also require the involvement of GMS. In this sense, it would be important to know which GMS can have the most influence on FMS so that a possible motor intervention program is more efficient and more likely to be successful.

For future research, we suggest the identification of instruments to assess fine motor skills in preschool children, with characteristics that allow their administration by the educator in the classroom context.

## Author contributions

PFl: conceptualization and writing draft preparation. PFl, EC, MM-C, and PFo: methodology and research. PFl, MM-C, and PFo: formal analysis. EC, MM-C, and PFo: writing revision and editing and supervision. All authors have read and agreed to the version of the manuscript.

## Funding

This work was supported by national funds (FCT—Portuguese Foundation for Science and Technology) under the project UIBD/DTP/04045/2020.

## Conflict of interest

The authors declare that the research was conducted in the absence of any commercial or financial relationships that could be construed as a potential conflict of interest.

## Publisher’s note

All claims expressed in this article are solely those of the authors and do not necessarily represent those of their affiliated organizations, or those of the publisher, the editors and the reviewers. Any product that may be evaluated in this article, or claim that may be made by its manufacturer, is not guaranteed or endorsed by the publisher.
